# Clusters of SARS-CoV-2 Infection Among Elementary School Educators and Students in One School District — Georgia, December 2020–January 2021

**DOI:** 10.15585/mmwr.mm7008e4

**Published:** 2021-02-26

**Authors:** Jeremy A. W. Gold, Jenna R. Gettings, Anne Kimball, Rachel Franklin, Grant Rivera, Elana Morris, Colleen Scott, Paula L. Marcet, Marisa Hast, Megan Swanson, Jazmyn McCloud, Lemlem Mehari, Ebony S. Thomas, Hannah L. Kirking, Jacqueline E. Tate, Janet Memark, Cherie Drenzek, Snigdha Vallabhaneni, Georgia K–12 School COVID-19 Investigation Team, Georgia K–12 School COVID-19 Investigation Team, Olivia Almendares, Abirami Balajee, Eleanor Burnett, Rebecca J. Chancey, Deanna Crosby, Morgane Donadel, Catherine Espinosa, Mary E. Evans, Katherine Fleming-Dutra, Catalina Forero, Kaitlin Forsberg, Esther Kukielka, Kiren Mitruka, Jasmine Y. Nakayama, Yoshinori Nakazawa, Michelle O’Hegarty, Emeka Oraka, Caroline Pratt, Marion E. Rice, Gurleen Roberts, Roxana Rodriguez Stewart, Raquel Sabogal, Emanny Sanchez, Andres Velasco-Villa, Mark K. Weng

**Affiliations:** ^1^CDC COVID-19 Response Team; ^2^Epidemic Intelligence Service, CDC; ^3^Georgia Department of Public Health; ^4^Cobb & Douglas Public Health, Marietta, Georgia; ^5^Marietta City Schools, Marietta, Georgia.; CDC; CDC, Maximus; CDC; CDC; Cobb & Douglas Public Health; CDC; CDC; CDC; CDC; CDC; CDC; CDC; CDC; CDC; CDC; CDC; CDC; General Dynamics Information Technology; CDC; CDC; Cobb & Douglas Public Health; CDC; CDC; CDC; CDC; CDC.

In-person learning benefits children and communities (*1*). Understanding the context in which transmission of SARS-CoV-2, the virus that causes coronavirus disease 2019 (COVID-19), occurs in schools is critical to improving the safety of in-person learning. During December 1, 2020–January 22, 2021, Cobb and Douglas Public Health (CDPH), the Georgia Department of Public Health (GDPH), and CDC investigated SARS-CoV-2 transmission in eight public elementary schools in a single school district. COVID-19 cases* among educators and students were either self-reported or identified by local public health officials. Close contacts (contacts)^†^ of persons with a COVID-19 case received testing. Among contacts who received positive test results, public health investigators assessed epidemiologic links, probable transmission directionality, and the likelihood of in-school transmission.^§^ Nine clusters of three or more epidemiologically linked COVID-19 cases were identified involving 13 educators and 32 students at six of the eight elementary schools. Two clusters involved probable educator-to-educator transmission that was followed by educator-to-student transmission and resulted in approximately one half (15 of 31) of school-associated cases. Sixty-nine household members of persons with school-associated cases were tested, and 18 (26%) received positive results. All nine transmission clusters involved less than ideal physical distancing, and five involved inadequate mask use by students. Educators were central to in-school transmission networks. Multifaceted mitigation measures in schools, including promotion of COVID-19 precautions outside of school, minimizing in-person adult interactions at school, and ensuring universal and correct mask use and physical distancing among educators and students when in-person interaction is unavoidable, are important in preventing in-school transmission of SARS-CoV-2. Although not required for reopening schools, COVID-19 vaccination should be considered as an additional mitigation measure to be added when available.

During the investigation period, which included 24 in-person school days during December 1, 2020–January 22, 2021, approximately 2,600 students (approximately 80% of the district’s elementary school students) and 700 staff members attended elementary school in person. During this period, COVID-19 incidence (7-day moving average number of cases per 100,000 persons) in Cobb County, Georgia, increased almost 300%, from 152 to 577 cases.^¶^ COVID-19 cases among educators and students attending in-person school were either self-reported to the school district or identified by local public health officials through laboratory results. Contacts who were exposed to persons with COVID-19 in school were identified by school officials, advised to quarantine based on local health department guidelines,** and referred to the investigation team.

Reverse transcription–polymerase chain reaction (RT-PCR) testing^††^ of anterior nasal swab specimens was offered free of charge to all contacts who were exposed in school, within 5–10 days of their last documented in-school exposure; 60% of identified contacts received testing, and 40% either declined testing or could not be reached. Semistructured virtual interviews with parents, educators, and principals were conducted to characterize the settings in which transmission likely occurred. Interviews included a review of symptom onset dates; possible exposures to persons with COVID-19 outside of school; and information on seating charts, classroom layouts, physical distancing, and compliance with recommended mask use during specific classroom interactions. Public health investigators visited four of six schools where SARS-CoV-2 transmission had been identified to observe adherence to recommended mitigation strategies and provide technical assistance. For contacts who received positive test results, epidemiologic links, probable transmission directionality, and the likelihood of in-school transmission were assessed by using interview data, testing dates, and symptom onset dates. Clusters were defined as epidemiologic links between an index patient and two or more persons who likely acquired SARS-CoV-2 infection in school (i.e., school-associated cases). Two contacts with positive test results were excluded because they likely acquired SARS-CoV-2 from household members outside of school. Household members of persons with school-associated cases were offered free RT-PCR testing. This activity was reviewed by CDPH, GDPH, and CDC and was conducted consistent with applicable Georgia law, federal law, and CDC policy.^§§^

During the investigation period, nine clusters of COVID-19 cases were identified, involving 13 educators and 32 students at six of the eight investigated elementary schools (Figure). The median cluster size, including household members, was six persons (range = 3–16). An educator was the index patient in four clusters (B, E, F, and I), a student was the index patient in one cluster (H), and in four clusters (A, C, D, and G), whether the index patient was the student, the educator, or both (i.e., two index cases occurred) could not be determined. Eight clusters (all except H) involved at least one educator and probable educator-to-student transmission. Four clusters (A, D, G, and H) involved probable student-to-student transmission, and three (A, C, and D) involved probable student-to-educator transmission. Two clusters (F and I) involved probable educator-to-educator transmission during in-person meetings or lunches, which was followed by educator-to-student transmission in the classroom and resulted in 15 of 31 (48%) school-associated cases. Sixty-nine household members of persons with school-associated cases were tested, and 18 (26%) received positive results. 

**FIGURE:**
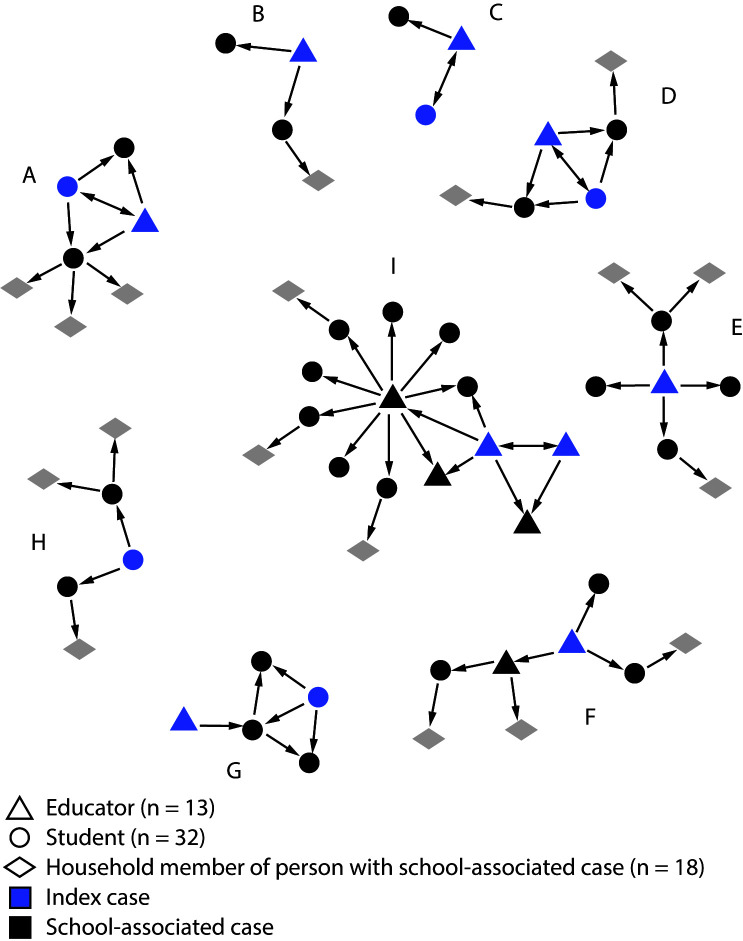
Nine SARS-CoV-2 transmission clusters (A–I)* at six elementary schools in one school district — Georgia, December 2020–January 2021 * The presence of two index cases within a cluster indicates that the index patient could not be determined or that two index patients might have occurred. Arrows indicate epidemiologic links between cases and probable transmission direction, determined by in-depth interviews of persons with cases, exposures outside of school, and symptom onset data.

Public health investigators identified several COVID-19 mitigation challenges. Although plastic dividers were placed on desks between students, students sat <3 ft apart. Physical distancing of >6 ft was not possible because of the high number of in-person students and classroom layouts. In seven clusters (A, B, C, D, E, F, and I), transmission among educators and students might have occurred during small group instruction sessions in which educators worked in close proximity to students. The school district mandated in-classroom mask use except while eating, and both reported and observed compliance during site visits was high. However, information obtained during interviews indicated that specific instances involving lack of or inadequate mask use by students likely contributed to spread in five clusters (A, C, E, G, and I). Students ate lunch in their classrooms, which might have facilitated spread. Opportunities to decrease nonessential in-person interactions among staff members during lesson planning and lunches were noted.

## Discussion

These findings suggest that educators can play an important role in in-school transmission and that in-school transmission can occur when physical distancing and mask compliance are not optimal. Previous investigations in other U.S. school districts found that low transmission rates in schools can be maintained in the setting of high community incidence (*2*,*3*). To ensure safer in-person learning during the COVID-19 pandemic, schools should implement multicomponent mitigation strategies, including efforts to prevent infection among educators, and promoting consistent, correct mask use and physical distancing wherever possible, especially during mealtime when masks are not being worn. 

The finding that educators play an important role in in-school transmission is consistent with findings from other investigations. A large prospective study of SARS-CoV-2 transmission in schools in the United Kingdom found that the most common type of transmission event was from educator to educator (*4*); in another large prospective study of transmission in German schools, in-school transmission rates were three times higher when the index case occurred in an educator than when the index case occurred in a student.^¶¶^ Measures to prevent SARS-CoV-2 infection among educators, including promotion of COVID-19 precautions outside of school, minimizing in-person adult interactions at school, ensuring mask compliance and physical distancing among educators when in-person interaction is unavoidable, and COVID-19 vaccination, when available, will likely reduce in-school transmission, particularly if implemented in a multifaceted approach. Messaging to improve awareness among educators about the risk for acquiring SARS-CoV-2 infections from colleagues in addition to students is needed. The school district has already implemented many of these measures, including administrative changes to prevent nonessential in-person interactions among educators.

The findings in this report are subject to at least three limitations. First, distinguishing in-school transmission from community transmission was challenging, particularly when the 7-day community incidence exceeded 150 cases per 100,000 persons and was increasing. Second, certain clusters and cases within clusters might not have been detected because not all contacts received testing. Finally, because adults with SARS-CoV-2 infection are more likely to have symptoms and be tested (*5*), index cases might have been more frequently identified in educators than in students, possibly resulting in missed instances of student-to-student and student-to-educator transmission.

Consistent with findings from international studies, this report found that initial infections among educators played a substantial role in in-school SARS-CoV-2 transmission and subsequent chains of infection to other educators, students, and households, highlighting the importance of preventing infections among educators in particular. Preventing SARS-CoV-2 infections in educators and students through multifaceted school mitigation measures is a critical component of preventing in-school transmission. Although not a requirement for reopening schools, adding COVID-19 vaccination for educators as an additional mitigation measure, when available, might serve several important functions, including protecting educators at risk for severe COVID-19–associated illness (*6*), potentially reducing in-school SARS-CoV-2 transmission, and minimizing interruptions to in-person learning, all of which have important implications for educational equity and community health. Because most children are not yet eligible for vaccination, continued implementation of multifaceted COVID-19 mitigation strategies in schools, including universal and correct mask use and physical distancing, even after educators are vaccinated, will be critical given the limited available evidence on reduction of transmission postvaccination and vaccine-related long-term protection (*7*).

**Summary**

**What is already known about this topic?**

In-person learning provides important benefits to children and communities. Understanding SARS-CoV-2 transmission in schools is critical to improving the safety of in-person learning.

**What is added by this report?**

An investigation of SARS-CoV-2 transmission in a Georgia school district during December 1, 2020–January 22, 2021, identified nine clusters of COVID-19 cases involving 13 educators and 32 students at six elementary schools. Two clusters involved probable educator-to-educator transmission that was followed by educator-to-student transmission in classrooms and resulted in approximately one half (15 of 31) of school-associated cases.

**What are the implications for public health practice?**

Educators might play a central role in in-school transmission networks. Preventing SARS-CoV-2 infections through multifaceted school mitigation measures and COVID-19 vaccination of educators is a critical component of preventing in-school transmission.

## Footnotes

* A COVID-19 case was defined as a positive SARS-CoV-2 reverse transcription–polymerase chain reaction or antigen test result in a person who attended school in person.

† Close contacts were defined as persons exposed to an index patient at school within 6 ft for >15 minutes per day during a 24-hour period while the index patient was infectious (48 hours before to 10 days after symptom onset or, if asymptomatic, 48 hours before to 10 days after specimen collection).

§ To be classified as having a school-associated COVID-19 case, a person had to meet three criteria: 1) the timing of symptom onset (if symptoms were present) and testing must have been consistent with acquisition of SARS-CoV-2 infection from the index patient or a person with a school-associated case in the school setting based on the known incubation period, 2) the person must have had close contact at school with the school index patient or another person with a school-associated case according to GDPH guidelines and during that patient’s infectious period, and 3) the person must not have had known community or household contact with anyone with confirmed COVID-19 in the 2 weeks before receiving a positive test result, including with the index patient or another person with a school-associated case outside of school.

¶ Incidence was calculated as a 7-day moving average per 100,000 persons and included persons with SARS-CoV-2 infection confirmed by reverse transcription–polymerase chain reaction or antigen testing.

** Students and staff members exposed to a COVID-19 patient were advised to quarantine for a minimum of 7 days if a specimen collected ≥5 days after exposure was negative for SARS-CoV-2 and they remained asymptomatic or for 10 days if they were not tested and remained asymptomatic. Persons with positive SARS-CoV-2 test results were advised to self-isolate for a minimum of 10 days after their positive test date or date of first symptom onset. https://dph.georgia.gov/contact; https://dph.georgia.gov/isolation-contact (accessed February 17, 2021)

†† Testing was performed at the Georgia Public Health Laboratory using the PerkinElmer COVID assay (Extraction-Chemagic, PCR-7500FastDx).

§§ 45 C.F.R. part 46, 21 C.F.R. part 56; 42 U.S.C. Sect. 241(d); 5 U.S.C. Sect. 552a; 44 U.S.C. Sect. 3501 et seq.

¶¶
https://www.medrxiv.org/content/10.1101/2021.02.04.21250670v1
