# *Erratum:* Vol. 70, No. 8

**DOI:** 10.15585/mmwr.mm7010a4

**Published:** 2021-03-12

In the report “Clusters of SARS-CoV-2 Infection Among Elementary School Educators and Students in One School District — Georgia, December 2020–January 2021,” on page 289, in the first full paragraph of the second column, the second sentence should have read “During this period, COVID-19 incidence (7-day **cumulative number of new cases** per 100,000 persons) in Cobb County, Georgia, increased almost 300%, from **194** to **704** cases.” On the same page, the ^¶^ footnote should have read “Incidence was calculated as a 7-day **cumulative incidence** per 100,000 persons **using date reported** and included persons with SARS-CoV-2 infection confirmed by reverse transcription–polymerase chain reaction or antigen testing.”

